# Development of a Positive Youth Development Program: Helping Parents to Improve Their Parenting Skills

**DOI:** 10.1100/tsw.2006.76

**Published:** 2006-03-28

**Authors:** Daniel T.L. Shek, Britta M. Lee

**Affiliations:** Social Welfare Practice and Research Centre, Department of Social Work, Chinese University of Hong Kong, Shatin, Hong Kong

**Keywords:** positive youth development, adolescence, Chinese, parents, parenting skills, Hong Kong

## Abstract

The Project P.A.T.H.S. (Positive Adolescent Training through Holistic Social Programs) is a positive youth development program that attempts to promote holistic development in adolescents in Hong Kong. In the Tier 2 Program of this project, social workers are expected to develop positive youth development programs for adolescents having greater psychosocial needs. They are required to submit proposals that will be evaluated in terms of whether the proposals are evidence based, and appropriate evaluation mechanisms are included. With reference to the literature on parental control processes that Chinese parents may be loose in their behavioral control and they tend to overemphasize academic excellence, it is argued that improvement of the parenting skills of parents of Chinese adolescents is an important area to be addressed. To facilitate social workers to prepare the related proposals, a sample proposal on how to improve the parenting skills of Chinese parents is described, including its conceptual framework, proposed program, and evaluation plan. It is argued that this supportive approach (i.e., preparation of a sample proposal) can help social workers to develop quality proposals on positive youth development programs in Hong Kong.

## INTRODUCTION

The Project P.A.T.H.S. (Positive Adolescent Training through Holistic Social Programs) is a positive youth development program designed to promote holistic adolescent development in Hong Kong, which is funded by the Hong Kong Jockey Club Charities Trust[1,2,3]. There are two tiers of programs (Tier 1 and Tier 2 Programs) in this project. The Tier 1 Program is a universal positive youth development program, where students in Secondary 1 to Secondary 3 will participate in the program, with 20 h of training in the school year at each grade comprising both core and elective programs. Because research findings suggested that roughly one-fifth of adolescents would need help of a deeper nature, the Tier 2 Program will generally be provided for at least one-fifth of the students who have greater psychosocial needs at each grade. Social welfare agencies providing school social work service joining the project are required to implement both the Tier 1 and Tier 2 Programs.

## SPECIAL PSYCHOSOCIAL NEEDS OF ADOLESCENTS

The Tier 2 Program (Selective Program) targets adolescents with greater psychosocial needs who are identified in the Tier 1 Program and/or via other sources. It is recommended that information based on multiple sources, including objective assessment tools (e.g., Family Assessment Instrument, Life Satisfaction Scale, Hong Kong Student Information Form), teachers' ratings, student records, and other relevant quantitative and qualitative information based on systematic assessment, will be used to identify students for the Tier 2 Program. In addition, it is suggested that particular attention should be paid to students with special needs in the academic, personal (e.g., adjustment, mental health, and value concerns), interpersonal, and family domains. As a general guideline, it is expected that at least one-fifth of the adolescents and/or the parents of the Tier 1 participants will participate in the Tier 2 Program.

For students who are identified as having special needs, they and/or their families will receive services based on the Tier 2 Program. The NGO applicants have the choice of designing appropriate programs that target the needs of the students with reference to the positive youth development constructs, goals, and objectives covered in this project. Several nonmutually exclusive examples for the Tier 2 Program include: (1) mentorship programs involving the alumni of the schools, (2) mental health promotion programs, (3) adventure-based counseling, (4) parenting programs, (5) service learning programs, and (6) resilience enhancement programs.

There are several required sections in the proposal for the Tier 2 Program. They include: (1) title of the program; (2) aims; (3) objectives; (4) proposed program to achieve the project objectives, including the conceptual bases of the intervention model, the rationale for using the model, and the evidence supporting the effectiveness of the proposed approach; and (5) proposed implementation details of the program, including the potential service targets of the participants and the proposed content of the program. The applicants are also required to specify the proposed evaluation mechanisms, including:

- Required evaluation mechanisms and related information: the number of participants to be served, the number of programs to be delivered, and the mechanisms to assess (1) the perceptions of the participants regarding the program and the worker(s) and (2) the participants' changes after joining the program.
- Additional evaluation mechanisms: the applicant may propose any other evaluation mechanisms to evaluate the program (e.g., qualitative evaluation and evaluation based on experimental approach).

## EVIDENCE-BASED POSITIVE YOUTH DEVELOPMENT PROGRAMS

To ensure that the funds are used in an appropriate manner and that the proposed projects are quality proposals, school social work service units will be invited to develop the Tier 2 Program with reference to the needs of the students and the positive youth development constructs covered in the program. The related proposals will be considered by the Social Welfare Department with the assistance of the Research Team. Two basic principles are used to assess the proposed project. First, the submitted proposal should be evidence based. The term "evidence based" is used in the broadest sense in this context[4,5]. It means that the proposed program should be based on: (1) well-articulated theories and/or well-argued conceptual frameworks and (2) available evidence showing that the proposed project has a promise in addressing the needs of the clients. As far as evidence is concerned, it refers to: (1) published and unpublished intervention research findings in local and foreign contexts and/or (2) properly documented past-practice experiences showing that the proposed program works. For innovative projects where direct evidence for the effectiveness of the proposed approach may be little, a greater weight will be put on the elegance of the conceptual model and the related evidence. The basic emphasis here is that we should have “knowledge-based practice” and “evidence-based practice” rather than solely “authority-based practice” or “worker-based practice”.

## ROLE OF EVALUATION

The second principle is that the proposal should include an evaluation plan. On completion of the program, the applicants must carry out the following three evaluation tasks: (1) to assess whether the number of programs delivered, as well as the number of service targets served, conform to the required output standards; (2) to assess the participants’ evaluation of the program and the worker(s); and (3) to assess the changes in the participants after joining the program. Actually, applicants are encouraged to devise different mechanisms to evaluate the proposed project and it is up to the applicants to propose what evaluation mechanisms will be used (including quantitative evaluation, qualitative evaluation, mixed-methods evaluation, or even constructionist and postmodern evaluation), provided that sufficient justifications are given.

## DISCUSSION

Shek, Lam, and Tsoi[5] pointed out that evidence-based social work practice was still in its infancy in Hong Kong and that social workers generally had a phobia about evaluation of social work programs. In addition, they pointed out that training may be inadequate on evaluation of social welfare programs provided for social workers. Against this background, it is the belief of the Research Team that the preparation of some sample proposals to guide social workers would facilitate their application procedures. One of the sample proposals on how parenting skills can be improved is described in this paper.

Based on a sample of students randomly drawn from the secondary student population in Hong Kong, Shek and Lee[6] pointed out that: (1) a significant proportion of Chinese parents did not exercise behavioral control over the social domain of their children; (2) some parents were high in their psychological control; (3) relative to the social domain, parents generally exerted more behavioral control in the academic domain of their children; and (4) roughly one-fourth of the respondents indicated that they were home alone or stayed with their friends without adult supervision after school. Generally speaking, these findings suggest that there is a need to improve the parenting skills of some Chinese parents. Shek[7] further showed that the parenting and parent-child relational processes in adolescents experiencing economic disadvantage were poorer than those in adolescents without economic disadvantage.

In short, the above research findings suggest that parental control processes play an important role in positive youth developmental outcomes. These findings are consistent with the assertions of the family ecological models that adolescent behavior is a function of dyadic parent-adolescent relationships and parental control processes. Obviously, improvement of parenting skills can be regarded as a prerequisite of promotion of bonding as a positive youth development construct. In the Appendix of this paper, a sample proposal on the development, implementation, and evaluation of a program that attempts to promote parenting effectiveness is described. It is our belief that this sample proposal can help colleagues in the social work and education fields to cultivate more confidence to develop proposals on positive youth development programs. This sample proposal is included in the Guidance Notes on Applications for the P.A.T.H.S. Project as an illustration of the support provided to the applicants.

## ACKNOWLEDGMENTS

The preparation for this work was financially supported by the Hong Kong Jockey Club Charities Trust, the Research Grants Council of the Government of the Hong Kong Special Administrative Region, Hong Kong (Grant CUHK4293/03H), and Wofoo Foundation.

## BIOSKETCHES

**Daniel T.L. Shek, PhD, FHKPs.S, BBS, JP**, is professor of Social Work in the Department of Social Work, the Chinese University of Hong Kong. He is on the editorial board of several scientific journals and has published extensively in international journals on social work, Chinese and English language, mental health, well-being, quality of life, adolescence, disadvantaged families and drug abuse. E-mail: DANIELSHEK@CUHK.EDU.HK. Website: http://www.cuhk.edu.hk/swk

**Britta M. Lee, MDes**, Social Welfare Practice and Research Centre, Department of Social Work, The Chinese University of Hong Kong, Hong Kong. E-mail: bmlee@swk.cuhk.edu.hk

## APPENDIX

A proposal on the development of a positive youth development program based on the application form for Project P.A.T.H.S. (Positive Adolescent Training through Holistic Social Programs) funded by the Hong Kong Jockey Club Charities Trust.

**1. Proposed program title**

Systematic Training for Effective Parenting Program (STEP) — Parents Group

**2. Aims of the program**

**2.1 Program aims**

To help parents learn effective ways to relate to their children and change dysfunctional relationships with their children

**2.2 Coverage of the 15 Positive Youth Development Constructs by the program**

Please copy this "☑" sign to the appropriate box(es).

☑ Promotion of Bonding
☐ Cultivation of Resilience
☐ Promotion of Social Competence
☐ Promotion of Emotional Competence
☐ Promotion of Cognitive Competence
☐ Promotion of Behavioral Competence
☐ Promotion of Moral Competence
☐ Cultivation of Self-Determination
☐ Promotion of Spirituality
☐ Development of Self-Efficacy
☐ Development of a Clear and Positive Identity
☐ Promotion of Beliefs in the Future
☐ Providing Recognition for Positive Behavior
☐ Providing Opportunities for Prosocial Involvement
☐ Fostering Prosocial Norms

**3. Specific objectives to be achieved**

1. To promote parenting knowledge and skills in the program participants
2. To help program participants develop a positive attitude to parenting
3. To reduce conflict between the parents and their children
4. To improve communication between the parents and their children

**4. Proposed intervention model to achieve the project objectives**

The present project will utilize the Systematic Training for Effective Parenting (STEP) Model (Dinkmeyer and McKay, 1983, 1989; Dinkmeyer, McKay, and Dinkmeyer, 1997a, 1997b; Dinkmeyer, McKay, McKay, and Dinkmeyer, 1998) as the guiding framework to run parenting groups for parents. Because there are cross-cultural differences in parenting behavior and beliefs, adaptation of the program content to suit the local scene and additional sessions will be designed. Construction of indigenous materials will also be attempted.

**4.1 Conceptual bases of the intervention model**

The conceptual bases of the STEP are closely related to the work of Alfred Adler and Rodolf Dreikurs (Dinkmeyer, Dinkmeyer, and Sperry, 1987; Dinkmeyer and McKay, 1983, 1989; Dinkmeyer, McKay, and Dinkmeyer, 1997a, 1997b; Dinkmeyer, McKay, McKay, and Dinkmeyer, 1998). The general assertions and principles in the STEP model are as follows:

- "To belong" is the basic social purpose that underlies all human behavior and human beings are primarily motivated by social connectedness. When one has a sense of belonging, one can face and deal with one's problems.
- Human beings are not seen in a mechanistic, passive, and reductionistic manner. Instead, human beings are regarded as creative, proactive, and holistic; who have the capacity to interpret, influence, and create events (Corey, 2001).
- The subjective frame of reference of an individual is emphasized.
- The focus of the intervention process includes provision of information, teaching, guiding, and offering encouragement to the clients (Corey, 2001). The relationship between the worker and the client is a collaborative and egalitarian one.
- There are five major influences on lifestyles, including heredity (e.g., temperament and intelligence), family atmosphere and values, role models, parenting, and position in the family.
- There are four goals of misbehavior (attention, power, revenge, and display of inadequacy) – adolescents misbehave because they want to achieve something. Therefore, it is important to understand the child's goals in misbehavior.
- It is also important to check one's responses to a child's misbehavior. There are differences between "good" parents and "responsible" parents.
- There are differences between "praise" and "encouragement" in parenting.
- It is important to explore alternatives and express parents' ideas and feelings to children.
- There are differences between natural and logical consequences of children's behavior.
- It is important to have family meetings.
- It is important for parents to develop confidence and use their potentials.

**4.2 Rationales for using the model**

There are several arguments for choosing the STEP as a practice model for parent education:

1. The model is based on the theories of Adler and Dreikurs.
2. The model is conceptually simple and easy to apply.
3. The model and the related programs have been utilized by over 3 million parents (Dinkmeyer, McKay, and Dinkmeyer, 1997b).
4. The model is the most widely chosen parent education model used by helping professionals in different fields (Ritchie and Partin, 1994).
5. Program manuals and training materials (e.g., videos) are available.
6. There are research findings showing that the proposed intervention was effective in Western contexts.
7. There are research findings showing that the proposed intervention was effective in the Hong Kong contexts (Ip, 2006; Kwok, 1994).

**4.3 Evidence supporting the effectiveness of the proposed approach**

- Williams, Omizo, and Abrams (1984) showed that parents participating in the STEP Program became more accepting and trusting than did the control subjects.
- Dinkmeyer, McKay, and Dinkmeyer (1990) presented research findings refuting the claim that the STEP Program was not effective.
- Allan (1994) reported that parents in the STEP Program found the I-messages and reflective listening techniques to be useful and they perceived the program to be effective.
- Snow, Kern, and Penick (1997) reported that in child and adolescent psychiatric treatment settings, the patients stayed longer in the treatment program and the therapeutic benefits were enhanced when their parents joined the STEP Program.
- Adams (2001) found that participants who completed the parent training reported more improvement in family functioning than did participants in the control group.
- Robinson, Robinson, and Dunn (2003) reviewed the research on the STEP and concluded that more evidence was needed to demonstrate the effectiveness of the program.

- In short, there are research findings supporting the effectiveness of the proposed approach, although the related findings are mixed.

**5. Proposed implementation details of the program**

**5.1 Potential service targets of the program**

Parents who report having parenting difficulties and parents who wish to improve their parenting will be recruited. The parents will be identified in the Tier 1 Program and via other sources (e.g., referral of the school social worker and publicity via PTA).

**5.2 Proposed content of the program**

- Three parenting groups (n = 32) with 8–12 parents in each group will be held.
- Pregroup interviews will be conducted to clarify mutual expectations with the parents.
- There will be ten 2-h sessions with the following proposed content:

| Session | Proposed Content |
|---|---|
| 1 | Overview of the program: STEP principles and philosophies; self-reflection on being a Chinese parent in Hong Kong; Chinese cultural beliefs about parents and parenting; assumptions about children and parents. |
| 2 | Understanding children’s behavior and misbehavior: goals of misbehavior and goals of positive behavior. |
| 3 | Understanding oneself as a parent: meaning of “good” parents in the Chinese culture; differences between “good” parents and “responsible” parents. |
| 4 | Building the child’s confidence and feelings of worth via encouragement: differences between praise and encouragement; Chinese views on praise and encouragement. |
| 5 | Communication with children: expression of feeling; reflective listening; emotional expression in Chinese adolescents and their families. |
| 6 | Exploring alternatives and expression of ideas and feelings to children: decisions for effective communication, developing the courage to be imperfect; emotional expression in Chinese parents. |
| 7 | Principles of natural and logical consequences: major differences between punishment and logical consequences; Chinese views about punishment and the related reasoning. |
| 8 | Applying natural and logical consequences in real-life settings: common conflict issues and resolution strategies in the Hong Kong context; real life challenges and success of applying the knowledge gained. |
| 9 | The family meeting: essentials, principles and mistakes of family meeting; beliefs and obstacles about using family meetings in Chinese families. |
| 10 | Developing parental confidence and using parent’s potentials: challenges faced by parents; parent’s self-defeating beliefs, ineffective parental responses, and effective actions; parenting efficacy in the Chinese culture; summary of the program. |

**6. Proposed evaluation mechanisms**

The required output standards and the requirement of implementing evaluation mechanisms to assess (1) perceptions of the participants regarding the program and the worker(s) and (2) the participants’ changes after joining the program, will be fulfilled accordingly (see Section 6.1). Besides, it is proposed that an additional evaluation mechanism will be used to triangulate the evaluation data obtained from different methods (see Section 6.2).

**6.1 Required evaluation mechanisms**

**A. Number of programs and participants —** Because there are 160 Secondary 1 students in the school, a total of 32 parents will be recruited to participate in three parenting groups (i.e., one-fifth of the students in Secondary 1), with ten 2-h sessions in each group.

For those who have missed some of the sessions (e.g., sickness), intensive make-up sessions will be provided.

B. **Evaluation of the program by the participants —** Each participant will be invited to respond to a self-administered subjective outcome evaluation form designed by the Research Team after completion of the program that assesses their satisfaction with the program and the worker. For each session, each member will also be invited to fill out a brief client satisfaction form designed by the worker at the end of the meeting. The form will be modeled after existing client satisfaction evaluation forms used in the agency.

C. **Changes in the program participants —** The participants' perceptions of their changes after joining the program will be assessed by the subjective outcome evaluation form designed by the Research Team as described in Part B above. Besides, objective outcome evaluation will also be used to assess changes in the program participants. For the objective outcome indicators, participants will respond to self-administered rating scales at pre- and post-test stages, assessing changes in knowledge and skills, attitudes, parent-adolescent conflict, and parent-adolescent communication:

**Objective 1 —** The questionnaire developed by Sharpley and Pointer (1980) will be used to assess the parents' acquisition of knowledge and skills introduced in the program. The Chinese version of this 20-item questionnaire was used by Kwok (1994) to assess the effectiveness of the STEP Program in Hong Kong.

**Objective 2 —** An abridged version of the Hereford Parent Attitude Survey (Hereford, 1963) will be used to assess parental attitudes in the following areas: confidence in parental role, causation of child's behavior, acceptance of the child's behavior and feelings, mutual understanding, and mutual trust. Kwok (1994) showed that the Chinese version of the scale was reliable in Chinese parents. An abridged version with 20 items will be used in this project.

**Objective 3 —** A 10-item measure of parent-adolescent conflict modeled after the Chinese version of the Conflict Behavior Questionnaire (Robin and Foster, 1989; Shek, 2002a) will be developed to assess parent-adolescent conflict.

Regarding the assessment of parent-adolescent communication (**Objective 4**), an assessment tool with 10 items will be developed after reviewing the relevant literature (Shek, 2001, 2002b). Furthermore, face validity of the instrument will be assessed by gathering feedback from experts in the field regarding the conceptual and applied relevance of the tool developed.

**6.2 Other evaluation mechanisms**

**Qualitative evaluation —** Toward the end of the program, the participants will be invited to write letters to their adolescent children, and their adolescent children will also be encouraged to write letters to their parents. Qualitative analyses extracting the major themes in the letters will give some ideas about the effectiveness of the program.

**7. Documentation of the evaluation findings**

A brief report utilizing the Report Template designed by the Research Team that documents the following areas will be submitted:

1. The achievement of output level (outlined in Section 6.1.A)
2. The participants' perception of the program and the worker(s)(outlined in Section 6.1.B)
3. Evaluation findings based on the subjective outcome evaluation and other evaluation mechanisms (outlined in Section 6.1.C and 6.2)
4. Brief description of the contents and implementation of the program
